# Driving with Intuition: A Preregistered Study about the EEG Anticipation of Simulated Random Car Accidents

**DOI:** 10.1371/journal.pone.0170370

**Published:** 2017-01-19

**Authors:** Gian Marco Duma, Giovanni Mento, Tommaso Manari, Massimiliano Martinelli, Patrizio Tressoldi

**Affiliations:** 1 Dipartimento di Psicologia Sociale e dello Sviluppo, Università di Padova, Padova, Italy; 2 Dipartimento di Psicologia Generale, Università di Padova, Padova, Italy; Canterbury Christ Church University, UNITED KINGDOM

## Abstract

The study of neural pre-stimulus or “anticipatory” activity opened a new window for understanding how the brain actively constructs the forthcoming reality. Usually, experimental paradigms designed to study anticipatory activity make use of stimuli. The purpose of the present study is to expand the study of neural anticipatory activity upon the temporal occurrence of dichotomic, statistically unpredictable (random) stimuli within an ecological experimental paradigm. To this purpose, we used a simplified driving simulation including two possible, randomly-presented trial types: a car crash end trial and a no car crash end trial. Event Related Potentials (ERP) were extracted -3,000 ms before stimulus onset. We identified a fronto-central negativity starting around 1,000 ms before car crash presentation. By contrast, a whole-scalp distributed positivity characterized the anticipatory activity observed before the end of the trial in the no car crash end condition. The present data are in line with the hypothesis that the brain may also anticipate dichotomic, statistically unpredictable stimuli, relaying onto different pre-stimulus ERP activity. Possible integration with car-smart-systems is also suggested.

## 1. Introduction

Our surrounding environment continuously stimulates our brain via multiple sensory modalities. However, full processing of all environmental stimuli would be extremely energy-consuming for our brain. In actual fact, when we observe the world, reality is not always chaotic and unpredictable. Rather, it shows some spatiotemporal regularities. The capacity to extract these regularities is a fundamental survival function for an organism, since it allows for the fine-grained, proactive control of resource allocation that is necessary for stimuli elaboration or action preparation. In other words, the brain takes advantage of these regularities, building up inner models of external reality, resulting in the possibility of anticipating a forthcoming event and consequently optimizing our behavior over time [1–4].

It has been suggested that the brain implements its predictive aptitude by capitalizing on a Bayesian computational architecture. This account posits the brain as a mechanism that makes continuous inferences about forthcoming stimuli on the basis of conditional probabilistic computations [5,6]. A possible way through which this can be accomplished is by exploiting the sensory contingencies provided by the external sensory environment itself.

Crucially, over the last decade, neuroimaging evidence has shown that the possibility of predicting ‘what’, ‘when’ and ‘where’ about forthcoming events, translates into anticipatory neural activity [3,4,7,8]. As a consequence of this, in the last decade the focus of cognitive neuroscience research has been progressively shifting from the investigation of post-stimulus neural activity to the exploration of the neurocognitive mechanisms taking place before the actual occurrence of a stimulus. The experimental evidence accumulated so far has provided fundamental knowledge that brain functioning can be conceived as an active constructor of reality, rather than as a simple, passive processor of external stimuli. One of the most used experimental paradigms to investigate anticipatory activity involves the study of an Event Related Potential (ERP) known as Contingent Negative Variation (CNV). The CNV is a slow cortical response of negative polarity, reflecting both expectancy and motor preparatory processes, that precedes anticipated events [8,9]. Remarkably, this ERP is considered as a reliable electrophysiological hallmark of timing, since its morphological features, including peak and slope inversion latency, mirror the duration of a previously encoded target interval when it has to be processed in temporal reproduction, discrimination or bisection tasks [8,10–12]. In the time-frequency domain, the findings reveal that anticipating sensory events resets the phase of slow delta-theta (2–8 Hz) activity before the stimulus occurs speeding up stimulus detection [13,14]. Evidence of the influence of anticipatory activity on stimuli elaboration has also been found in the alpha (8-12Hz) and beta (13-30Hz) oscillatory range. In fact, the desynchronization of alpha oscillation has been correlated with time of prediction of the anticipated event’s occurrence [15]. The general interpretation of alpha desynchronization is that it is an active inhibitory mechanism that regulates sensory information processing, optimizing the elaboration of task-relevant elements [16]. On the other hand, beta oscillations are generally associated with motor preparation, which facilitate the action execution required from the forthcoming stimulus [17].

### 1.2 Anticipatory ERP activity preceding unpredictable events

Some researchers have posed the question as to whether this anticipatory activity may still be observed in the context of statistically unpredictable (random) events. From an adaptive point of view, these situations are clearly more complicated and potentially threatening for life. Hence, the possibility for biological organisms to even approximately anticipate these events would represent a clear adaptive advantage. Usually, the experimental paradigms employed in these kinds of studies imply dichotomic stimuli, leading researchers to test the hypothesis that an anticipatory modulatory effect may discriminate between them. The first result was presented by Levin et al. [18], who observed a larger CNV before subject responses to target stimuli presented in a randomized sequence. More recently, research has provided further evidence of differential pre-stimulus cortical activity prior to unpredictable stimuli. Radin, Vieten, Michel, & Delorme [19], tested a group of meditators, showing significant differences in pre-stimulus cortical activity before a randomly presented light flash or tone, highlighting the similarities with Stimulus Preceding Negativity, and hypothesizing that “*SPN may be a marker not only for conventional forms of anticipation*, *but potentially for retrocausal forms as well*” (pag. 295). A noteworthy consideration is that anticipatory activity before unpredictable stimuli in randomized presentations showed similarities to the activity observed before predictable stimuli, suggesting that similar underlying neurocognitive mechanisms may subserve them both. Yet, it is still to be fully understood whether the capacity to anticipate unpredictable stimuli might be extended to an ecological context. To this purpose, we created a simplified driving simulation paradigm, which may include a car accident (i.e., ‘crash end’ condition) or not (i.e., ‘no crash end’ condition). The experimental design entailed a passive and an active task. In the *passive task*, participants had to simply and passively observe a car moving along the street and mentally anticipate the temporal occurrence of the car crash however, they were unable to explicitly act so as to avoid its occurrence. On the other hand, in the *active task*, they were asked to try to avoid the car accident by withdrawing the accelerator (spacebar) at the exact moment in which they felt that a car accident was occurring. This allowed us to examine the ERP activity arising before each of the two possible events (i.e., crash or no-crash end) in order to test the hypothesis that the brain activity might present “anticipatory” effects. The experimental paradigm was purposely designed with the future aim of integrating human-machine smart systems based on the possibility of using ERP anticipatory activity as a neural marker of upcoming, potentially threatening events. Before such high-tech approaches are reached, it is important to test for the presence of a reliable brain anticipatory activity preceding unpredictable stimuli. Here we borrow the definition of this pre-stimulus activity from Mossbridge et al. [20] who named it Predictive Psychophysiological Anticipatory Activity (PPAA). PPAA is a category including different type of signals, e.g. EEG, ECG, etc., which refers to pre-stimulus onset activity. We used this definition in order to place our study within this theoretical framework.

## 2. Material and Methods

### 2.1 Study preregistration

The study was preregistered on Open Science Framework (https://osf.io/rcjfa), following the guidelines of the Preregistration Challenge (https://cos.io/prereg).

### 2.2 Participants

As declared in the pre-registration format, our study included 40 participants from a total of 47 participants initially recruited through an online announcement. All the participants were students from different faculties of the University of Padova. All participants were rewarded with 10€ for their participation in the study. They had no history of neurological, neuropsychiatric disorders or drug consumption and had normal or corrected-to-normal visual acuity. After being informed of the conditions of the study, all participants signed a voluntary consent form, and they were told that they could stop participating in the experiment at any time. The study was approved by the ethical committee of the School of Psychology of the University of Padova (id. protocol 1930). We used a 20% threshold of rejected epochs as an inclusion criterion to the final dataset. At the end of data collection, we rejected 7 participants over 47 as they showed artifact-contamination of more than 20%. We performed a full analysis on 40 participants, 12 males and 28 females, mean age = 22.9; SD = 2.1.

### 2.3 Study design

**Fig 1:**
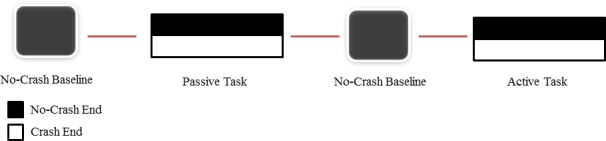
Experimental design. Passive or Active Task blocks were always presented after the Baseline condition. Blocks presentation was randomized between subjects. No-Crash End and Crash End conditions were randomized both in the Active and Passive Task.

We used a simplified driving simulation as behavioral task. To this purpose, a graphic interface was specifically designed by one of the co-authors (MM), who programmed the driving-simulation software environment using the open source software ‘Unreal® Engine4’ on a PC running Windows 10. The simulation was presented on a 21.5” monitor with a screen resolution of 1920 ×1080 pixels and a driving-simulation resolution of 1280 × 720. The experiment was divided into a passive and an active task. A third, baseline task was further administered (see Fig 1). All participants underwent all the experimental tasks.

#### 2.3.1 Baseline task

**Fig 2:**
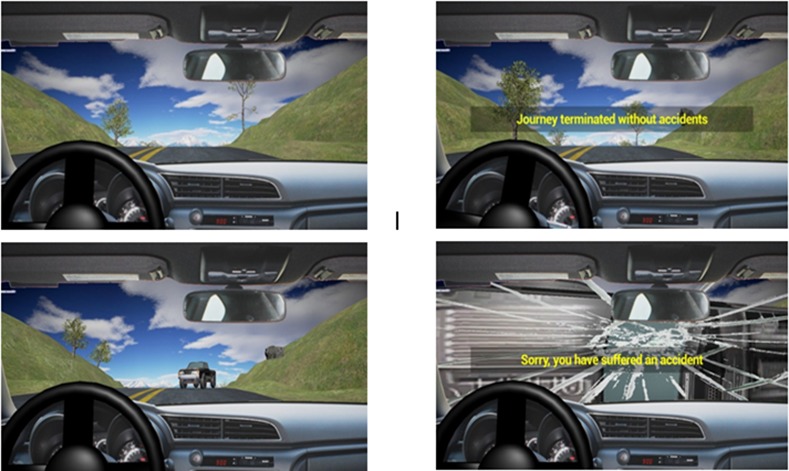
a) First person perspective of the car moving along the street in all the conditions of the “passive task”; b) Trial ending in ‘No Crash Baseline‘ and ‘No Crash End’ condition; c) Car presentation in ‘Crash End’; d) Trial ending with a car crash in ‘Crash End’ condition.

In this task, participants were asked to passively watch the driving simulation, which consisted of a total of 14 trials. Each trial lasted from 7 to 10 s, with the whole task lasting about 120s. This consisted of a car moving along the street from a first-person perspective (see Fig 2A). The baseline task had two aims. On the one hand, it was administered for participants to familiarize themselves with the driving simulation environment. On the other hand, all participants were explicitly told that in this task no car accidents would occur in any trial. This information induced participants to establish certainty about what to expect to occur at the end of each run. As a further consideration, although the duration of each trial was jittered (i.e., between 7 and 10 s) in order to avoid habituation, the relatively short length allowed participants to implicitly establish temporal expectancy about the putative end of each trial [8]. This explorative choice allowed us to use this condition as a baseline for detecting the anticipatory neural activity induced by the explicit instruction of no car accident as well as by the implicit timing about the end of the trial due to the quasi-regular temporal structure of the trials [21].

#### 2.3.2 Passive task

In the passive task. participants had to passively watch the driving simulation, as in the baseline condition. Yet, in this case they were explicitly told that each trial could end with the simulation of a car accident (‘crash end’) or not (‘no crash end’). The administration of these two experimental conditions were randomized both within and between subject. Specifically, the crash end condition consisted in a video simulating a street accident with a car coming from the opposite direction, together with a crash sound (see Fig 2C and 2D). By contrast, in the no crash end condition, the sentence “Journey terminated without accidents” appeared on the screen (see Fig 2B) at the end of the trial. All participants received the following instructions: ‘You will see a car moving along the street, just like you did in the baseline task. However, in the present task a car crash could occur. Importantly, if and when the car crash will be presented is totally random. Your task is to try to mentally anticipate the car presentation’. In order to avoid any implicit or explicit prediction about the timing of the trial end, the duration of each run was randomly varied between 25 and 40s. Overall, the car accident was present in 50% of trials, with the remaining trials being ‘no crash’ trials. A total of 20 trials was delivered for each condition. Crucially, this information was not given to the participants in order to prevent them from using any conscious strategy based on the prediction of future events by counting the number of the previous ones. The trial type sequence was randomized within and between participants by using the randomization algorithm implemented in the Unreal® Engine software, so that the occurrence of a ‘crash’ or ‘no crash’ trial was fully unpredictable. Crucially, neither visual nor auditory cues were presented to predict the accident.

#### 2.3.3 Active task

In the active task, all the experimental manipulations, including trial type and duration randomization, were kept equal as in the passive task. However, in contrast to the passive task, in this case participants were given the possibility of controlling the speed of the car by pressing (or withdrawing) the space bar, which acted as the car accelerator. Crucially, the speed control allowed participants to behaviorally avoid the car crash. In particular, if they decelerated within a ‘decision time’ range, lasting 5 seconds and randomly inserted (from 6 to 25 s), the car crash was avoided. Not only were participants not informed about the beginning or the duration of the ‘decision time’, but they were also unaware about the duration of the trial. As in the passive task, neither explicit nor implicit cues suggested the forthcoming event type. The general instruction given to participants was to decelerate just once per trial, that is, only when they felt the ‘intuition’ that an upcoming car crash was going to occur. Any attempts at deceleration outside the decision time window were codified as ‘false positive’.

The Active task was primarily included because we wanted to obtain a behavioral correlate of the anticipatory brain activity, in line with the hypothesis that this anticipation could influence participants’ behavior. As a noteworthy consideration, a possible confound for the investigation of the anticipatory ERP activity in the active task may derive from the presence of a motor preparation/execution electrophysiological activity (i.e., pressing or withdrawing the space bar). In fact, this may result in the elicitation of a response-preceding readiness potential (RP), which may overlap any activity related to car crash anticipation by itself [8].

### 2.4 EEG recording

During the entire session, the electroencephalogram (EEG) was continuously acquired using a Micromed: SD MRI 64 system (Micromed/Treviso, Italy), amplified and digitized with sampling frequency of 512 Hz. A 32-channel Electro-Cup International montage was employed, in accordance with 10–20 international system [22], referenced to the bilateral linked mastoids, using Ag/AgCl electrodes. All electrode impedances were less than 10 KΩ and balanced. Eye movements were recorded using two electrical-oculogram (EOG), placed at the outer canthus of each eye, respectively. The raw data of all included and excluded participants are available here: https://figshare.com/articles/Driving_with_Intuition_raw_data/3573888.

### 2.5 Data analysis

The exploratory analyses will be explicitly declared in order to distinguish them from the planned (preregistered) ones.

#### 2.5.1 Active task: behavioural analysis

For each participant, the rate of crash skipping (hits) were extracted and codified as ‘True accident Prediction’, while the deceleration outside the decision time window (false positive) were codified as ‘False accident Prediction’. Finally, the trials in which the car accident was not present and the participant did not decelerate, were codified as ‘True No Accident Prediction’. We tested the hypothesis that the number of predictions was above chance level considering both ‘True accident’ and ‘True No Accident’. No ERP analyses were conducted for the Active task, since, in this case the possible presence of a PPAA may overlap with RP activity.

#### 2.5.2 Passive task: ERP analysis

All EEG recordings were processed offline using the MATLAB toolbox EEGLAB [22]. The data were first band-pass filtered between 0.1 and 30 Hz and segmented into epochs starting –3000 ms before stimulus onset and ending 500 ms after it. Epochs were then visually inspected to interpolate bad channels and remove rare artifacts. Artifact-reduced data were then subjected to Independent Component Analysis [23]. All independent components were visually inspected, and only those related to eye blinks or eye movements according to morphology and scalp distribution were discarded. The remaining components were then projected back to the electrode space to obtain cleaner EEG epochs. The remaining epochs containing excessive noise or drift (± 100 μV at any electrode) were further rejected. Seven participants out of 47, showing excessive noisy signal, were discarded. In the remaining 40 participants, the total number of artifact-free trials per condition was: Baseline (mean = 13.53; SD = 0.71), No Crash End Passive Task (mean = 19.37; SD = 0.86) and Crash End Passive Task (mean = 19.25, SD = 1.08). Subject average and grand average ERPs were generated for each electrode site and experimental condition. Statistical differences between conditions were calculated by using both the Mass Univariate Analysis toolbox [24] and the Fieldtrip Brainstorm toolbox [25]. ERP components were statistically compared in pairs of experimental conditions by performing cluster-based, lower-tailed t-tests. A non-parametric, 1000 random permutation test was applied as multiple-comparison correction. For the Mass Univariate Analysis we reported p-value, critical t-test score and correspondent alpha-wise level, whereas Fieldtrip toolbox analyses [26] we reported p-value, where cluster metric corresponds to the sum of all the t-values of all the data points in the cluster and cluster size corresponds to the number of data points included in each cluster (the sum of the number of signals in the cluster over all the time points). We applied a lower-tailed t-test because we hypothesized that the anticipatory effect (the PPAA) could be included in the negative slow wave classification related to anticipatory neural activity, such as CNV or SPN. As an additional statistical analysis, the mean voltage amplitude for each subject and over all electrodes was measured and extracted (-1000 ms time window) performing Bayesian parameters analysis estimating the effect size with the corresponding High Density Intervals (HDIs) of the different comparisons using the BEST package [27] and BayesFactors (BF) with a Cauchy prior = .20 using the JASP software [28].

#### 2.5.3 Explorative analyses: time on task effect

To deepen the understanding of anticipatory brain activity, we decided to explore its dynamic evolution across the task, known as Time on Task Effect (TOT; [8]). Addressing the presence of TOT is important because it provides a dynamic picture of changes in anticipatory brain activity over time, allowing us to make further inferences about attentional resource allocation. More specifically, as previously reported [8], the dynamic evolution of anticipatory ERP activity may unveil the presence of implicit learning effects. As a matter of fact, the use of a randomized experimental design should abolish TOT implicit learning effects, allowing to control for any possible bias in the trials condition distribution over time. The epoched ERP dataset relative to each task of interest (i.e., Baseline, Crash End Passive Task and No Crash End Passive Task) was clustered into 3 consecutive temporal bins, each one including one third of the trials total number. The three temporal bins were hence defined as the early, the middle and the late phase of the task. The trials within each phase were then averaged and statistically compared using the same pair-wise statistical comparisons applied for conventional ERP analysis. The statistical comparisons were performed both *within* and *between* tasks. More specifically: ‘early *vs* middle’, ‘early *vs* late’ and ‘middle *vs* late’ comparisons were done *within* each mentioned task. Furthermore, we examined TOT *between* the Passive and Active task. This was done by calculating, for each experimental task, three difference waves, obtained from the following TOT subtractions: ‘early minus middle’, ‘early minus late’ and ‘middle minus late’. The resulting difference waves were then compared between tasks as follows: the ‘early minus late’ difference wave relative to the No Crash Baseline *vs*. the ‘early minus late’ difference wave relative to the Crash End Passive Task and so on.

#### 2.5.4 Explorative analyses: spectral density analysis

While ERP provides time-domain electrophysiological information, in order to perform a more complete exploration of anticipatory brain activity, we decided to perform an explorative spectral density analysis using Welch’s method. Our purpose was to examine the power of the different frequencies before stimulus onset, comparing the three “passive” conditions. First, we performed the power spectrum density analysis with Welch’s method implemented in Brainstorm, analysing the signal in a time window including 1000 ms before the stimulus onset (i.e., -1000 to 0 ms). A window overlap ratio of 50% was used and the delta, theta, alpha and beta frequency bands were targeted. We did not extract either gamma 1 or gamma 2 because a band-pass filter between 0.1 and 30Hz was originally applied to analyze ERP activity, this excluded high-frequency data from the dataset used to calculate the power spectrum. Subsequently, we performed statistical comparisons *between* tasks by using the same approach employed for conventional and TOT ERP analysis but, in this case, separately for each frequency band.

### 2.6 Results

#### 2.6.1 Active task: behavioural results

As shown in Table 1, there was no statistical evidence of above chance performance (equal to 10), either for the ‘True accident prediction’ or for the ‘True no accident prediction’.

**Table 1:** Descriptive and inferential statistics relative to behavioural performance in the active task.

	Mean (SD)	One-sample t-test	Cohen’s *d*	BF_10_
**True AccPrediction**	10.9 (3.7)	1.5	.25	1.7
**True NoAccPrediction**	8.1 (4.6)	-2.5	-.39	.17

#### 2.6.2 Passive task: ERP results

**Fig 3:**
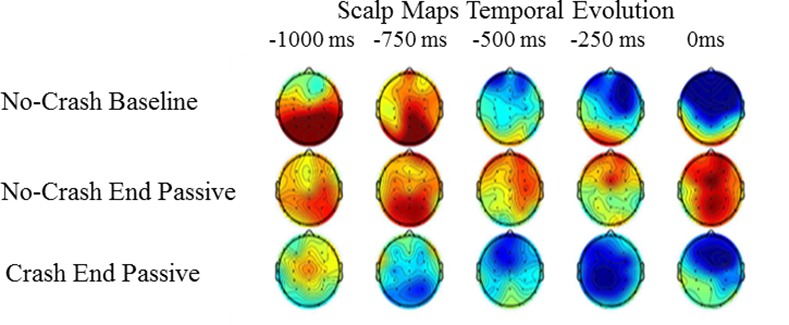
Topographical distribution of temporal evolution of the brain anticipatory activity in the Baseline and the two passive conditions.

From the visual inspection of ERP morphology, we noticed that the tasks started to differentiate each other at around -1000 ms before stimulus onset, while no appreciable differences were present before this point. To be sure that eventual ERP modulations did not occur before the interval in which the difference was actually observed, we performed pair-wise statistical analyses by contrasting the mean ERP amplitude relative to the time window from -3000 ms to -1000 ms, measured in the Baseline, Crash End Passive and No-Crash End Passive conditions. Specifically, 1000-permutation cluster-based, two-tailed analyses, using both Mass univariate toolbox and Field trip brainbox toolbox implemented in the Brainstorm software, were performed. Since these analyses did not yield significant differences, we concluded that the ERP signal between -3000 ms and -1000 ms did not differ between tasks. Hence, we considered a baseline from -1500 ms to -1000 ms before stimulus onset, including a -500 ms interval before the beginning of the observed ERP difference. The visual inspection of the scalp map distribution (see Fig 3) further revealed that, while the ‘Crash End Passive’ and the ‘Baseline’ conditions were characterized by a negative activity distributed over the fronto-central electrodes, the ‘No Crash End Passive’ condition did not show the same pattern.

**Fig 4:**
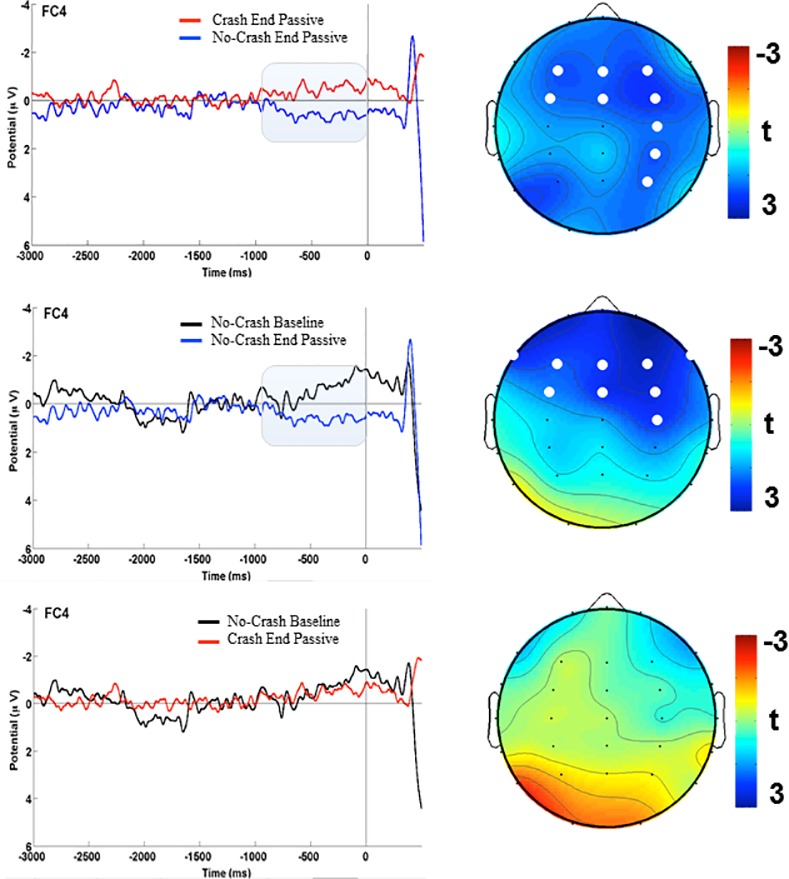
Event Related Potentials of the pre-stimulus activity are represented on the left side of the figure. The time windows of analyses are highlighted. The topographical distributions of t-values are represented on right side of the figure. The white dots are the electrodes where the statistically significant differences are expressed. 4a) ‘Crash End’ and ‘No Crash End’,4b) ‘No Crash Baseline’ and ‘No Crash End’, 4c) ‘No Crash Baseline” and ‘Crash End’.

The statistics confirmed the qualitative pattern showing greater negativity at ‘Crash End Passive’ compared to ‘No Crash End Passive’ conditions (*p* = 0.03, critical t-score for each contrast was -1.81, corresponding to a 0.03 test-wise alpha level). The statistical analysis yielded findings in line with the observed electrical scalp-map distribution, since it identified a spatiotemporal cluster showing more negative voltages at the following electrodes: F3, FZ, F4, C4, P4, FC3, FC4, CP4, FCz (Fig 4A). These results are consistent with previous studies [3,4,8,29]. Interestingly, we also found a significant difference between the ‘No Crash Baseline’ and the ‘No Crash End Passive’ condition (*p* = 0.03, critical t-score = -1.69 that corresponds to a test-wise alpha level of 0.04), the difference being expressed at following electrodes: F7, F3, FZ, F4, F8, C4, FC3, FC4, FCz (Fig 4B).

No significant differences between the ‘No Crash Baseline’ and the ‘Crash End Passive’ were found (see Fig 4C).

These findings were further confirmed by the Bayesian analyses. The results are presented in Table 2.

**Table 2:** Effect sizes with their corresponding 95% HDIs and BayesFactor values, of the comparisons between the mean amplitudes in the three conditions.

	Baseline vs No Crash End	Baseline vs Crash End	Crash vs No Crash End
**Means (SD)**	-.18 (1.7) vs .36 (1.3)	-.18 (1.7) vs -.21 (1.04)	-.21 (1.04) vs .36(1.3)
**Effect size (95%HDIs)**	.47 (-.03 to .98)	.09 (-.55 to .41)	.44 (-.04 to .88)
**BF**_**10**_	2.02	.43	2.5

From the above results a clear difference emerges between the mean amplitude of the ‘No Crash Baseline’ and ‘Crash End Passive’ conditions with respect to the passive No Crash one. These differences are slightly superior using the average amplitude of the channels that emerged from the permutation analysis (see Table 3).

**Table 3:** Effect sizes with their corresponding 95% HDIs and BayesFactor values, of the comparisons between the three conditions using the channels emerged from the permutation analysis.

	Baseline vs No Crash End	Baseline vs Crash End	Crash vs No Crash End
**Means (SD)**	-.38 (2.09) vs .47 (1.7)	-.38 (2.09) vs -.36 (1.4)	-.36 (1.4) vs .47 (1.7)
**Effect size (95%HDIs)**	.51 (-.01 to .99)	.05 (-.50 to .40)	.52 (.06 to .98)
**BF**_**10**_	3.9	0.46	3.5

As it can be seen, both the statistical approaches did not reveal any significant difference between the ‘Baseline’ and the ‘Crash End Passive’ comparison when considering the mean ERP amplitude in the -1000 to 0 ms time window. However, a closer inspection of the ms-by-ms temporal unfolding of the anticipatory activity over the scalp showed a different spatio-temporal pattern between these two conditions. Specifically, in this temporal window, the negativity featuring both the ‘Crash End Passive’ and ‘Baseline’ emerged earlier in the first case, i.e., at about 750 ms before stimulus onset. By contrast, in the Baseline condition, the fronto-central negativity started later, i.e., at about 500 ms. Moreover, the morphological inspection of ERP activity taking place at about 200 ms before stimulus onset displayed a boost of anticipatory activity for the Baseline as compared to the Crash End Passive condition (Fig 3). Both these elements suggest that, although not statistically comparable, these two conditions may engage at least partially distinct neurocognitive mechanisms.

#### 2.6.3 Explorative analyses: time on task analysis results

**Fig 5:**
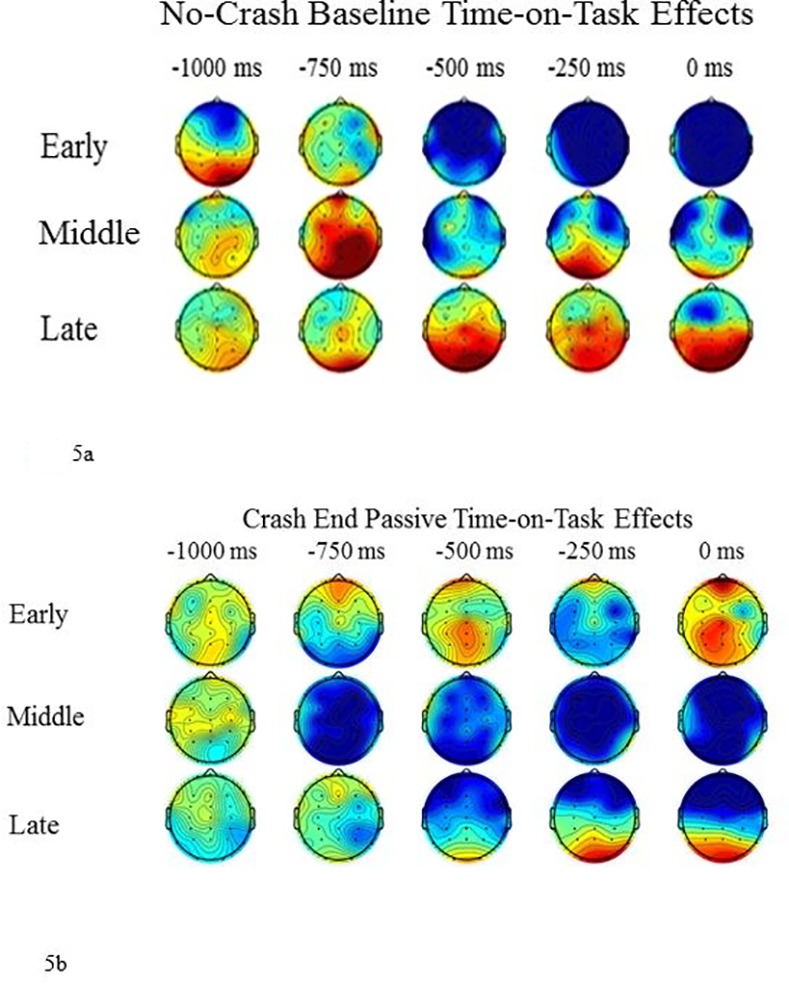
**a.** Topographic scalp distribution of the evolution of anticipatory activity of the “Baseline” condition in the three phases of the Time-on-Task analysis. **b.** Topographic scalp distribution of the evolution of anticipatory activity of the ‘Crash End Passive’ condition in the three phases of the Time-on-Task analysis.

No significant TOT effects were found either relatively to the ‘Crash End Passive’ or to the ‘No Crash End Passive’ conditions, confirming that, in both of these cases, the anticipatory ERP activity was not significantly modulated in amplitude across the task. Furthermore, the TOT did not present specific directional trends in either cases. It was in fact highly variable across trials, in early, middle and late phases (see Fig 5B and S1 Fig). This finding would suggest that no implicit learning effects are present either in the Crash End Passive or No Crash End conditions. However, we found a significant TOT difference with respect to the Baseline condition. In particular, we found more negative amplitude in the early as compared to the middle temporal bin [*p* = 0.045, Critical t-score = -2.021 that corresponds to a test-wise alpha level of 0.025; electrodes: C4, P4, T6, CP4, TP8] as well as in the early as compared to late temporal bin [*p* = 0.044, critical t-score = -1.874 that corresponds to a test-wise alpha level of 0.034; electrodes: F4, C4, P4, T6, FC4, CP4, TP8]. No significant differences were observed in the middle *vs*. late contrast. The within-condition TOT modulatory effect is shown in Fig 5A, which shows that the anticipatory ERP activity in the Baseline condition is at its maximum during the first trials but decreases over time, disappearing in the middle and late temporal bins. This peculiar pattern can be accounted for by the fact that, in highly temporally predictable tasks, participants are usually engaged in an implicit learning of the temporal contingency among stimuli. This translates into a higher allocation of neural resources over the first trials, when they are implicitly engaged in extracting the rule at the basis of such temporal contingency. Once this knowledge is acquired, there is a progressive disinvestment of resources in favor of the implementation of possible actions induced by temporal expectations that have been progressively established over time. This mechanism is automatic, since it requires neither explicit a priori temporal knowledge nor the active engagement in an experimental task, being already established in the first months of life [30,31]. In our paradigm, the baseline condition induced a strong expectancy about the end of the trial, due to the low variability of the temporal duration of each trial, which ranged between 7 and 10 sec. In other words, after few trials participants established a strong, implicit temporal expectancy about the approximate end of each run. Moreover, they had certainty about the condition as they were informed at the outset that, in the baseline condition, no car crash would occur. Taking together both these issues, it is not surprising that we observed strong anticipatory activity in the Baseline condition that, nevertheless, diminished dramatically in the middle and late phases of the task as a result of habituation. Moreover, it is important to mention that this activity trend is a distinctive element between Baseline and Crash End Passive, as revealed by between-task comparison. In fact, a significant difference was observed in ‘early *vs*. middle’ [*p* = 0.02, cluster metric = -31, cluster size = 28; significant differences at FZ, CZ, C3, C4, P3, PZ P4, O1, O2, CP3, CP4, FPZ, CPZ, FCZ electrodes] and ‘early *vs*. late’ comparisons [p = 0.0439, cluster metric = -16, cluster size = 16; significant differences at C3, CZ, C4, PZ, P4, CP3, CP4, CPZ electrodes]. These findings allowed us to further dissociate the ERP activity arising before the crash in the baseline and passive task, respectively, as reflecting at least partially different mechanisms. Indeed, it can be argued that in the baseline condition participants easily extracted the rule allowing them to implement anticipatory neural activity. We argue that this kind of anticipatory activity can be interpreted as a passive CNV component most probably induced by the regular statistical distribution of the temporal duration of trials in the baseline condition.

#### 2.6.4 Explorative analyses: spectral density results

**Fig 6:**
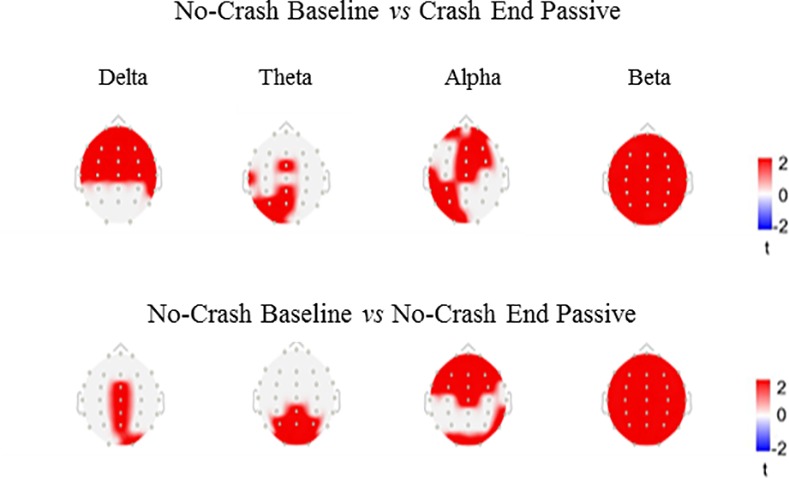
Statistically significant differences between power spectrum density in the comparison between No Crash Baseline vs Crash End Passive and No Crash Baseline vs No Crash Passive in the bands: Delta (2-4Hz), Theta (5-7Hz), Alpha (8-12Hz), Beta (15-29Hz).

As a further element, allowing for the dissociation of baseline and crash end conditions, we found statistical differences in their Welch power spectrum distribution (PSD) (*p* = 0.002, cluster metric = 350, cluster size = 218). Statistical significant differences are found also in ‘Baseline’ *vs* ‘No Crash End Passive’ (*p* = 0.002, cluster metric = 323, cluster size = 174). Fig 6 shows the scalp distribution of the significant differences. No differences in the power of frequency were found between ‘Crash End Passive’ and ‘No Crash End Passive’ conditions.

## 3 Discussion

The aim of the present study was to investigate whether anticipatory neural activity can be extended to randomized stimuli presented in a more ecological context. Although other authors already reported evidence of anticipatory neural activity preceding randomized, unpredictable stimuli [19], some criticisms about the methodological reliability of these study has been raised [32]. Our study aimed at investigating this apparently controversial phenomenon using a more robust methodological and statistical approach.

By measuring the EEG activity during the passive viewing of an ecological, driving simulation, we observed a fronto-central negative ERP activity, here interpreted as a PPAA, that arose before the occurrence of a statistically unpredictable, randomized car accident. Importantly, our findings show, for the first time, that this activity can be dissociated from the expectancy-related CNV wave that can arise on the basis of the implicit learning of the temporal contingency between stimuli as observed in the ‘Baseline’ condition [8]. This distinction stems from several sources of evidence. First, the PPAA and the CNV elicited in our study are characterized by a different temporal and spatial pattern. Second, the dynamic of the across-task evolution (TOT analysis) was different between the CNV and the PPAA, since the first one was larger at the beginning of the task while the second one did not show significant amplitude modulation over time. This different temporal pattern may be also explained by assuming a different level of sustained attention between the baseline and the passive task. In the first case, participants had the certainty that ‘relevant’ (i.e., crash) events would not have occurred. This may have induced a habituation of the neural anticipatory response from the early to the late phase of the task. By contrast, the presence of a possible salient event (a car crash) may have reduce or canceled out this effect. A third line of evidence derives from the Welch power spectrum density, which suggested that the anticipatory, oscillatory neuronal activity is differently modulated in the Baseline compared with the crash end condition. Taken together, these results suggest that the CNV and the PPAA are supported by at least partially distinct and dissociable neurocognitive mechanisms.

Simply providing statistically significant data is not enough to explain a phenomenon. In order to be fully understood, the data should be linked with a theoretical framework. A first, legitimate criticism arising when interpreting these results, stems from possible methodological issues. For example, it could be claimed that the observed anticipatory differences between the Passive Crash End and the Passive No Crash End conditions are simply due to methodological artifacts. In the attempt to address any possible methodological shortcomings of the present study, we considered several possible technical or design biases. Concerning the first aspect, we can exclude differences due to the frame’s screen refresh because no frame screen refreshed errors were detected, which could otherwise have been interpreted as a cue of the forthcoming stimulus. We can also exclude differences in the randomization procedure. The randomization procedure implemented by the Unreal® Engine4 software uses a pseudo random generator algorithm, which can be found on the web site https://www.unrealengine.com/what-is-unreal-engine-4. The random sequences of all 40 participants are made available here: https://figshare.com/s/8d43eac9f73bf7e29bf5. Concerning other possible biases, we asked whether the observed ERP difference may be simply due to a classical Gambler’s fallacy phenomenon in the passive task, that is, the expectation that a ‘Crash End’ probability will increase linearly with the number of the previous ‘No Crash End’ trials. A simulation by Mossbridge et al. [33], showed that with approximately 40 participants, and a random presentation of the two classes of events, this effect can be ruled out or it is very unlikely given that the expectation of a ‘Crash End’ event is balanced by the expectation of a ‘No Crash End’ one.

Our results could be inserted in the “predictive brain” perspective [34–36], extending the capability of the brain to use its inner model to predict future events including those based on the presentation of random stimuli. The capacity to predict environmental dynamics and prepare the body-system to react to incoming stimuli, allows for more efficient and adaptive behaviour, therefore, from an evolutionary point of view, to ‘anticipate’ a random stimulus could be the most adaptive capacity of the brain, improving the likelihood of the organism’s survival.

Some theoretical frameworks have been proposed to elucidate anticipatory effects. These explanations include the hypothesis that our brain could also show quantum-like temporal non-locality, similar to that observed in quantum physics. A noteworthy proposal is the Orchestrated Object Reduction (ORCH-OR) theory proposed by Hameroff and Penrose [37,38]. This theoretical explanation implicates microtubules as quantum processors in the brain. Interpretations of anticipatory activity, arising from this approach, involve explanations borrowed from quantum mechanics and modelled to help provide a psychologically relevant understanding. Hagan, Hameroff, & Tuszyński [39] showed how, despite the warm, noisy and wet intracellular milieu, quantum effects were present at the microtubule level. The superposition is assured, in the aromatic rings of the tubuline protein, by the London Forces. Despite these forces being 40 times weaker than a hydrogen bond, thanks to the high number of coupled electronic clouds, they can influence the protein conformation. The superposition state could be extended to the neighbouring cells via a tunneling-effect through the gap-junction. While the proteins are in the superposition state, in physical terms, they are in a *coherence* state, where all the possible states in which the system may collapse exist at the same time, meaning that the future system conformations also exist at the same time. In *coherence*, all the states are entangled with each other, therefore, it is possible that the present state is entangled with a future state, with an information exchange from the future to the present, implying retrocausal effects. The term retrocausal, refers to a condition in which the temporal causal links are inverted. Although there are, as yet, few experimental confirmations in psychology [40], in physics there is a vast literature about retrocausal effects in quantum mechanics [41,42]. The theoretical interpretation, reported here is a possible explanation of how the brain is capable of anticipating stimuli that are considered unpredictable. However, solving the conundrum of finding a substratum to retrocausal effects in psychology is not the purpose of the present research. The main purpose is to start to delineate the neurophysiologic identity and confines of ‘anticipation effects’, such as topographical scalp activation, latency and polarity with the purpose to investigate also the activation source (e.g. with a High Density-EEG), so that the contributions of other researchers, can help to draw a picture that is starting to become clearer. Defining the identity of this anticipation effect might have future applications in the field of human-machine integration. In fact, if we delineate the characteristics that compose pre-stimulus activity in random stimuli, a software with on-line analysis might be able to detect them. If we observe the potential evolution of smart-systems for cars, it is possible to imagine a machine-integrated EEG software. The opportunity to detect possible forthcoming dangers could be a life-saving element. With an EEG cup on the drivers’ head, the software could provide feedback when the characteristic elements of anticipatory activity are detected, providing the driver with feedback about a possible forthcoming danger. The present technology is not far from this possible goal. To develop this, three issues need to be addressed in an effort to clarify and improve our understanding. First, the neuroscience community should define the neurophysiological characteristics of anticipatory activity for randomly presented stimuli more precisely. The second step would be to improve EEG-on-line data analyses, and the last step should be focused on developing more effective and efficient ways to record live brain activity. The strength of this vision is its multidisciplinary approach, which includes neuroscience, informatics and engineering, providing an opportunity to nourish a fruitful research field.

## Supporting Information

S1 Fig Topographic scalp distribution of the evolution of anticipatory activity of the “No Crash End” condition in the three phases of the Time-on-Task analysis. (TIF)

## References

[1] Friston K. A theory of cortical responses. Philos Trans R Soc Lond B Biol Sci. 2005;360(1456):815–36. 10.1098/rstb.2005.1622 15937014 PMC1569488

[2] Nobre AC. Orienting attention to instants in time. Vol. 39, Neuropsychologia. 2001 p. 1317–28. 11566314 10.1016/s0028-3932(01)00120-8

[3] Mento G, Vallesi A. Spatiotemporally dissociable neural signatures for generating and updating expectation over time in children: A High Density-ERP study. Dev Cogn Neurosci. 2016;19:98–106. 10.1016/j.dcn.2016.02.008 26946428 PMC6988099

[4] Mento G, Tarantino V, Vallesi A, Bisiacchi PS. Spatiotemporal Neurodynamics Underlying Internally and Externally Driven Temporal Prediction: A High Spatial Resolution ERP Study. J Cogn Neurosci. 2015;27(3):425–39. 10.1162/jocn_a_00715 25203276

[5] Kleinschmidt A, Büchel C, Hutton C, Friston KJ, Frackowiak RSJ. The neural structures expressing perceptual hysteresis in visual letter recognition. Neuron. 2002;34(4):659–66. 12062048 10.1016/s0896-6273(02)00694-3

[6] Yuille AL, Bulthoff HH, Kersten D, Mamassian P. Perception as Bayesian Inference. Annu Rev Psychol. 1996;55:271–304. 10.1146/annurev.psych.55.090902.142005 14744217

[7] Cui H, Andersen R a. Different Representations of Potential and Selected Motor Plans by Distinct Parietal Areas. J Neurosci. 2011;31(49):18130–6. 10.1523/JNEUROSCI.6247-10.2011 22159124 PMC3327481

[8] Mento G. The passive CNV: carving out the contribution of task-related processes to expectancy. Front Hum Neurosci. 2013;7(December):827. 24376409 10.3389/fnhum.2013.00827 PMC3859886

[9] Walter WG, Cooper R, Aldridge VJ, McCallum WC, Winter A. L. Contingent Negative Variation: An Electric Sign of Sensori-Motor Association and Expectancy in the Human Brain. Nature. 1964;203(4943):380–4. 14197376 10.1038/203380a0

[10] Praamstra P. Electrophysiological markers of foreperiod effects In: Nobre AC, Coull JT, editors. Attention and time. Oxfrod University Press; 2010 pp.331–345.

[11] Macar F, Vidal F. The CNV peak: An index of decision making and temporal memory. Psychophysiology. 2003 11 1;40(6):950–4. 14986848 10.1111/1469-8986.00113

[12] Van Rijn H, Kononowicz TW, Meck WH, Ng KK, Penney TB. Contingent negative variation and its relation to time estimation: a theoretical evaluation. Front Integr Neurosci. 2011;5(December):1–5. 22207841 10.3389/fnint.2011.00091 PMC3246349

[13] Stefanics G, Hangya B, Hernádi I, Winkler I, Lakatos P, Ulbert I, et al Phase entrainment of human delta oscillations can mediate the effects of expectation on reaction speed. J Neurosci. 2010;30(41):13578–85. 10.1523/JNEUROSCI.0703-10.2010 20943899 PMC4427664

[14] Lakatos P, O’Connell MN, Barczak A, Mills A, Javitt DC, Schroeder CE. The Leading Sense: Supramodal Control of Neurophysiological Context by Attention. Neuron. 2009;64(3):419–30. 10.1016/j.neuron.2009.10.014 19914189 PMC2909660

[15] Mathewson K. Pulsed out of awareness: EEG alpha oscillations represent a pulsed inhibition of ongoing cortical processing. (Doctoral dissertation, University of Illinois at Urbana-Champaign). 2011. http://hdl.handle.net/2142/26295 10.3389/fpsyg.2011.00099 PMC3132674 21779257

[16] Jensen O, Bonnefond M, VanRullen R. An oscillatory mechanism for prioritizing salient unattended stimuli. Vol. 16, Trends in Cognitive Sciences. 2012 p. 200–5. 10.1016/j.tics.2012.03.002 22436764

[17] Jenkinson N, Brown P. New insights into the relationship between dopamine, beta oscillations and motor function. Vol. 34, Trends in Neurosciences. 2011 p. 611–8. 10.1016/j.tins.2011.09.003 22018805

[18] Levin J., Kennedy J. The relationship of slow cortical potentials to psi information—Google Scholar. J Parapsychol. 1975;25–6.

[19] Radin DI, Vieten C, Michel L, Delorme A. Electrocortical activity prior to unpredictable stimuli in meditators and nonmeditators. Explor J Sci Heal. 2011;7(5):286–99. 10.1016/j.explore.2011.06.004 21907152

[20] Mossbridge JA, Tressoldi P, Utts J, Ives JA, Radin D, Jonas WB. Predicting the unpredictable: critical analysis and practical implications of predictive anticipatory activity. Front Hum Neurosci. 2014 3 25; 8:146 10.3389/fnhum.2014.00146 24723870 PMC3971164

[21] Mento G, Tarantino V, Sarlo M, Bisiacchi PS. Automatic Temporal Expectancy: A High-Density Event-Related Potential Study. PLoS One. 2013;8(5). 10.1371/journal.pone.0062896 PMC3641105 23650537

[22] Klem GH, Otto Lu Èders H, Jasper H, Elger C. The ten±twenty electrode system of the International Federation. 10590970

[23] Delorme A, Makeig S. EEGLAB: An open source toolbox for analysis of single-trial EEG dynamics including independent component analysis. J Neurosci Methods. 2004;134(1):9–21. 10.1016/j.jneumeth.2003.10.009 15102499

[24] Stone J V, Stone J V. Independent component analysis: an introduction. Trends Cogn Sci. 2002;6(2):59–64. 15866182 10.1016/s1364-6613(00)01813-1

[25] Groppe DM, Urbach TP, Kutas M. Mass univariate analysis of event-related brain potentials/fields I: A critical tutorial review. Vol. 48, Psychophysiology. 2011 p. 1711–25. 10.1111/j.1469-8986.2011.01273.x 21895683 PMC4060794

[26] Tadel F, Baillet S, Mosher JC, Pantazis D, Leahy RM. Brainstorm: A user-friendly application for MEG/EEG analysis. Comput Intell Neurosci. 2011;2011. 10.1155/2011/879716 PMC3090754 21584256

[27] Oostenveld R, Fries P, Maris E, Schoffelen JM. FieldTrip: Open source software for advanced analysis of MEG, EEG, and invasive electrophysiological data. Comput Intell Neurosci. 2011;2011. 10.1155/2011/156869 PMC3021840 21253357

[28] Kruschke JK. Bayesian estimation supersedes the t test. J Exp Psychol Gen. 2013;142(2):573–603. 10.1037/a0029146 22774788

[29] Jasp Team. JASP (Version 0.7.5.5)[Computer software]. 2016.

[30] Miniussi C, Wilding EL, Coull JT, Nobre AC. Orienting attention in time. Modulation of brain potentials. Brain. 1999;122:1507–18. 10430834 10.1093/brain/122.8.1507

[31] Mento G., Valenza E. Spatiotemporal neurodynamics of temporal expectancy in infants and adults. Sci Report. 2016;6. 10.1038/srep36525 PMC5109914 27811953

[32] Schwarzkopf DS. We should have seen this coming. Front Hum Neurosci. 2014 5 27; 8:332 10.3389/fnhum.2014.00332 24904372 PMC4034337

[33] Mossbridge J, Tressoldi P, Utts J, Ives JA, Radin D, Jonas WB. We Did See This Coming: Response to, We Should Have Seen This Coming, by D. Sam Schwarzkopf. 2015 Jan 13; Available from: http://arxiv.org/abs/1501.03179

[34] Pezzulo G. Coordinating with the future: The anticipatory nature of representation. Minds Mach. 2008;18(2):179–225.

[35] Bar M. The proactive brain: using analogies and associations to generate predictions. Trends Cogn Sci. 2007;11(7):280–9. 10.1016/j.tics.2007.05.005 17548232

[36] Friston K. The free-energy principle: a unified brain theory? Nat Rev Neurosci. 2010;11(2):127–38. 10.1038/nrn2787 20068583

[37] Hameroff S. How quantum brain biology can rescue conscious free will. Front Integr Neurosci. 2012;6(October):1–17. 23091452 10.3389/fnint.2012.00093 PMC3470100

[38] Hameroff S, Penrose R. Consciousness in the universe: A review of the “Orch OR” theory. Phys Life Rev. 2014;11(1):39–78. 10.1016/j.plrev.2013.08.002 24070914

[39] Hagan S, Hameroff SR, Tuszyński JA. Quantum computation in brain microtubules: Decoherence and biological feasibility. Phys Rev E. 2002 6 10;65(6):61901. 10.1103/PhysRevE.65.061901 12188753

[40] Tressoldi PE, Maier MA, Buechner VL, Khrennikov A. A macroscopic violation of no-signaling in time inequalities? How to test temporal entanglement with behavioral observables. Front Psychol 2015 7 29. 10.3389/fpsyg.2015.01061 PMC4518645 26283993

[41] Reznik D, Aharonov J, Bell J, Bell J, Bell J, Bell J, et al Time-symmetric formulation of quantum mechanics. Phys Rev A. 1995 10;52(4):2538–50. 9912531 10.1103/physreva.52.2538

[42] Egg M. Delayed-Choice Experiments and the Metaphysics of Entanglement. Found Phys. 2013 9 21; 43(9):1124–35.

