# Supplementary figures and images for: Driving with Intuition: A Preregistered Study about the EEG Anticipation of Simulated Random Car Accidents
Source: PLoS One. 2017 Jan 19;12(1):e0170370. doi: 10.1371/journal.pone.0170370 (PMC5245833; doi:10.1371/journal.pone.0170370)

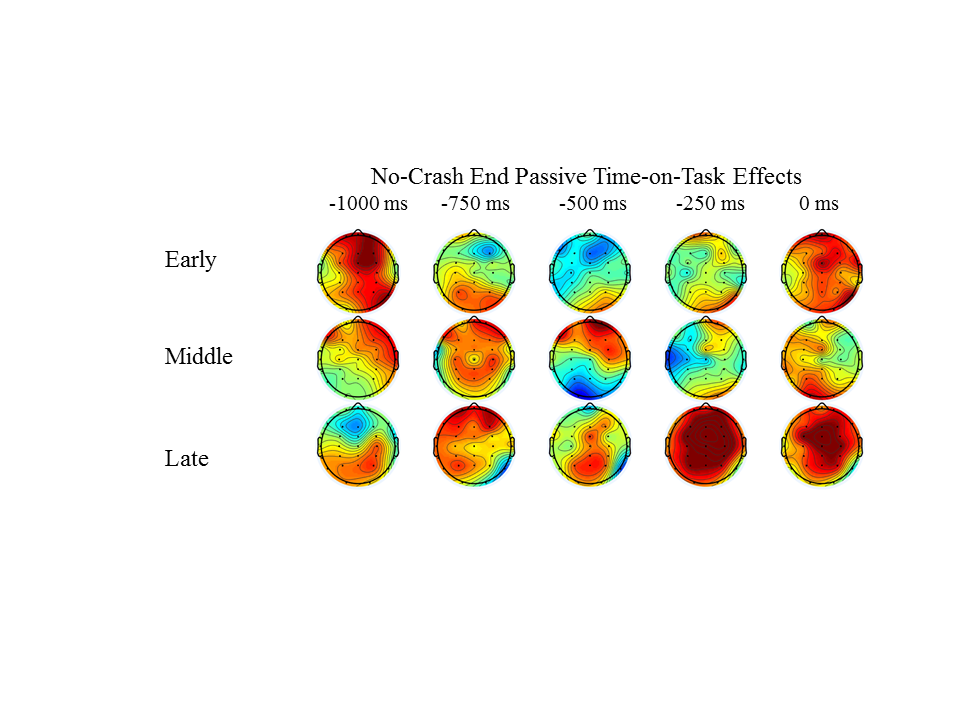

Supplement: S1 Fig — (TIF) [file pone.0170370.s001.tif]
